# Methodological Aspects of μLC-MS/MS for Wide-Scale Proteomic Analysis of Anthracycline-Induced Cardiomyopathy

**DOI:** 10.1021/acsomega.4c09377

**Published:** 2025-03-18

**Authors:** Rudolf Kupčík, Olga Lenčová, Yvona Mazurová, Martin Štěrba, Marie Vajrychová

**Affiliations:** 1 Biomedical Research Centre, University Hospital Hradec Králové, Hradec Králové 500 05, Czech Republic; 2 Department of Pharmacology, Faculty of Medicine in Hradec Králové, Charles University, Hradec Králové 500 03, Czech Republic

## Abstract

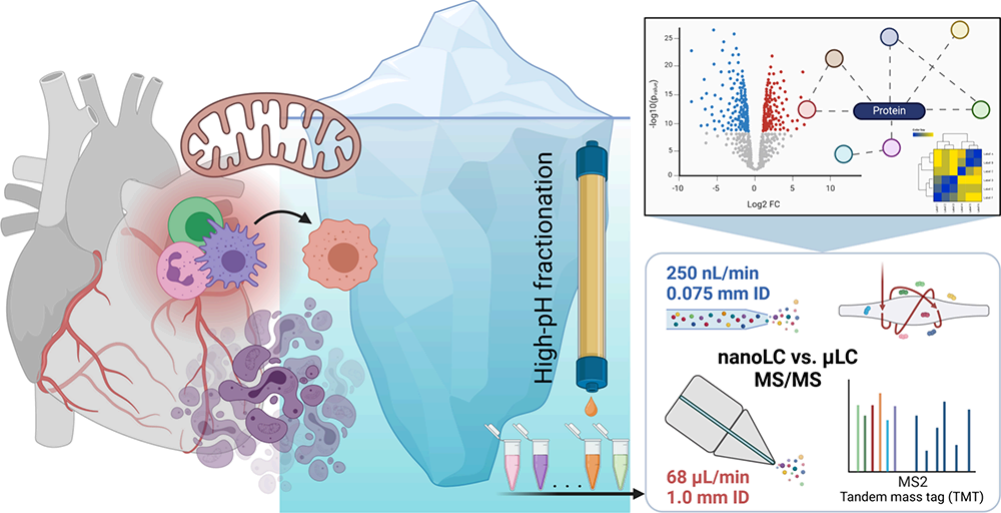

The efforts to utilize microflow liquid chromatography hyphenated to tandem mass spectrometry (μLC-MS/MS) for deep-scale proteomic analysis are still growing. In this work, two-dimensional LC separation and peptide derivatization by a tandem mass tag (TMT) were used to assess the capability of μLC-MS/MS to reveal protein changes associated with the severe chronic anthracycline cardiotoxicity phenotype in comparison with nanoflow liquid chromatography (nLC-MS/MS). The analysis of the control and anthracycline-treated rabbit myocardium by μLC-MS/MS and nLC-MS/MS allowed quantification of 3956 and 4549 proteins, respectively, with 84% of these proteins shared in both data sets. Both nLC-MS/MS and μLC-MS/MS revealed marked global proteome dysregulation in severe anthracycline cardiotoxicity, with a significant change in approximately 55% of all detected proteins. The μLC-MS/MS analysis allowed less compressed and more precise determination of the TMT channel ratio and correspondingly broader fold-change protein distribution than nLC-MS/MS. The total number of significantly changed proteins was higher in nLC-MS/MS (2498 vs 2183, 1900 proteins shared), whereas the opposite was true for a number of significantly changed proteins with a fold-change cutoff ≥ 2 (535 vs 820). The profound changes concerned mainly proteins of cardiomyocyte sarcomeres, costameres, intercalated discs, mitochondria, and extracellular matrix. In addition, distinct alterations in immune and defense response were found with a remarkable involvement of type I interferon signaling that has been recently hypothesized to be essential for anthracycline cardiotoxicity pathogenesis. Hence, μLC-MS/MS was found to be a sound alternative to nLC-MS/MS that can be useful for comprehensive mapping of global myocardial proteome alterations such as those associated with severe anthracycline cardiotoxicity.

## Introduction

In the past few years, μLC-MS/MS has become a reasonable alternative to the nLC-MS/MS setup for bottom-up proteomics. The limited sample loading, high sample complexity, wide dynamic concentration range of protein contents, and time-consuming LC gradients are factors challenging the performance of nanoelectrospray ionization. With new analytical columns with a larger inner diameter (ID) up to 1 mm, the speed and the sensitivity of new generation of mass spectrometers are transferring the bottom-up proteomics toward microflow LC operating in tens of μL/min. Recent high-impact articles showed an exceptional robustness with the excellent stability of RT across thousands of injections and extraordinary proteome coverage using a microflow LC setup.^1,2^ From initial attempts yielding less than 1000 proteins and few thousands of peptides,^3^ nearly 10,000 proteins and 130,000 peptides have been attained by coupling of microflow LC and modern hybrid Orbitrap mass spectrometers after 2020.^4^ Hence, μLC-MS/MS appears as a fully viable option for efficient and deep MS-based proteome research.

Cardiovascular (CVD) diseases are the most frequent cause of mortality in developed countries, followed by cancer in second place. Current anticancer treatments have dramatically improved the survival of patients with many different types of tumors, but often at the cost of a significant toxic burden on the host organism.^5−7^ As a consequence of anticancer therapy, long-term cancer survivors may suffer from markedly higher overall cardiovascular morbidity. For instance, repeated cycles of anthracycline (ANT) chemotherapy (e.g., daunorubicin/DAU/ and doxorubicin) may induce the development of irreversible cardiomyopathy and chronic heart failure. Hence, there is an unrelenting effort to advance the understanding to complex changes occurring in the heart during toxic damage induced by anticancer drugs.^8^

Proteomic research significantly contributed to advances that have been made in the field of cardiovascular medicine over past two decades including important insights into the mechanism of anticancer drug-induced cardiotoxicity in animal models.^9^ However, wealth of these data was acquired using two-dimensional (2D)-PAGE-MS/MS.^10−13^ Shotgun LC-MS/MS studies are still underrepresented and rather employed to anticancer-induced toxicity in cell cultures *in vitro*.^14−16^ Modern bottom-up LC-MS/MS studies performed on myocardial samples taken from translational animal models after long-term exposition to anticancer drugs are still scarce and provided only limited insights yet.^17^ Indeed, LC-MS/MS proteomics has been successfully exploited to research of a number of cardiac pathologies.^18−26^ In these studies, 1145–4168 myocardial proteins were detected using both 1D or 2D nLC-MS/MS and a distinct setup of protein quantification (LFQ/TMT/iTRAQ).

The aim of our study is to assess the suitability of μLC-MS/MS application for appropriate mapping of myocardial proteome changes associated with the development of severe cardiomyopathy induced by anthracycline anticancer therapy in a well-established rabbit model. We followed our previously published article reporting the μLC-MS/MS setup employing analytical column with ID of 1.0 mm^3^ and introduced it to quantitative TMT-based proteomic analysis of global myocardial proteome. Outcomes were compared to the background of the protein coverage achieved by parallel nLC-MS/MS analysis.

## Materials and Methods

### Reagents, Chemicals, and Drugs

All reagents and chemicals for tissue homogenization and protein digestion were purchased from Sigma-Aldrich (St. Louis, MO, USA) or Fisher Scientific (Hampton, NH, USA), if not specified otherwise. Organic solvents and water (LC-MS grade) were purchased from Honeywell (Morris Plains, NJ, USA) or Fisher Scientific, if not specified otherwise. Daunorubicin (DAU, hydrochloride salt, pharmaceutical grade) was purchased from Euroasia′s (India).

### Animal Model of Severe Anthracycline Cardiomyopathy

Adult, male New Zealand White rabbits (3.0–3.5 kg Velaz, Praha, Czech Republic) were caged individually under standard conditions with ad libitum access to a standard rabbit chow diet and tap water. The study was approved by the Animal Welfare Committee of Charles University, Faculty of Medicine in Hradec Králové. Chronic anthracycline cardiotoxicity was induced by the administration of daunorubicin (DAU, 3 mg/kg, i.v., weekly for 10 weeks). For the proteomic analysis, three animals fulfilling predefined criteria of severe cardiomyopathy and heart failure were included into the study, i.e., treatment-induced decline of the left ventricular (LV) systolic function from baseline at least by 1/3 as examined by echocardiography and apparent signs of blood congestion due to the heart failure observed during in necropsy (hydrothorax, increased lung weight and ascites). The severe cardiotoxic phenotype was deliberately selected as it was assumed to represent the most prominent alterations of the myocardial proteome (changes to cardiomyocytes but also other cells of the myocardium and extracellular matrix). Three animals receiving saline in the same volume instead of DAU in the same schedule were used as the controls. The LV systolic function was examined noninvasively under light anesthesia (ketamine 30 mg/kg; midazolam 1.25 mg/kg, i.m.) by echocardiography (Vivid 4, 10 MHz probe; GE Healthcare, Chicago, IL, USA) at the beginning and at the end of the experiment. LV fractional shortening (FS) and LV ejection fraction (EF) as parameters of systolic function were determined as described previously.^27^ At the scheduled end of experiment, LV catheterization examination was performed under surgical anesthesia (pentobarbital 10 mg/kg, i.v.) using a Mikro-Tip pressure catheter (2.3F; Millar Instruments, Houston, TX, USA) as described in detail elsewhere.^27^

### Collection of Myocardial Samples and Tissue Homogenization

A week after the last DAU dose and immediately after the scheduled invasive examination of cardiac function, the animals were overdosed with pentobarbital. During necropsy, the heart was rapidly excised, washed, and briefly retrogradely perfused with ice-cold saline using a syringe to flush out the blood. The transverse sections of the heart ventricles were obtained for histological examination. The rest of the LV myocardium was cut and then shock-frozen in liquid nitrogen. Frozen samples were pulverized in liquid nitrogen and stored at −80 °C until further analyses.

### Histological Examination of the Myocardium

Transverse sections of the heart ventricles were immersed in 4% neutral formaldehyde for 5–7 days and embedded in paraffin. Serial paraffin sections (5 μm thick) were stained with Masson’s blue trichrome. The preparations were scanned using SmartZoom ClassRoom software (Smart In Media AG, Germany), and photomicrographs were then taken at 3× and 20× magnification of the scans.

### Solubilization of the LV-Myocardial Homogenate

Approximately 50 mg of the homogenized LV myocardium was used for proteomic analysis. A solubilization buffer consisting of 1% sodium deoxycholate (SDC) in 8 M urea was added to each homogenized sample in the volume (μL) equal to 5-fold of sample weight (approximately 50 mg). All samples were kept on ice from this point on further. Samples were sonicated in an ultrasound bath for 15 min and incubated at 10 °C for 60 min under continuous shaking (1400 rpm). Due to high viscosity, a lysis buffer was added according to the equation described above. Samples were centrifuged at 10,000*g* (15 min, 10 °C), and supernatants were transferred into clean microtubes and kept at −80 °C. The total protein concentration was determined by bicinchoninic acid and copper sulfate solution (BCA protein assay kit) before further processing.

### Protein Digestion

After the tissue homogenization and lysis, 150 μg of each sample was transferred to the fresh tube and 1 M triethylammonium bicarbonate (TEAB, pH 8.5) and water was added to the samples to get final concentration of 0.1% SDC, 100 mM TEAB, and 0.8 M urea. Reversibly oxidized cysteines were reduced with 5 mM Tris(2-carboxyethyl)phosphine hydrochloride (TCEP) at 37 °C for 60 min. Free thiol groups were further alkylated by 10 mM *S*-methylmethanethiosulfonate (MMTS) at RT for 10 min. Subsequently, ice-cold acetone in the 6-fold volume was added to each sample, and proteins were precipitated by keeping samples at −20 °C for 4 h. All samples were then centrifuged at 8000*g* (10 min, 4 °C), supernatants were then carefully decanted, and pellets were shortly dried using vacuum concentrator to remove the possible remnants of acetone. Prepared pellets were redissolved in 100 mM TEAB/0.1% SDC and rLys-C (Wako Pure Chemical Corporation, Osaka, Japan) and sequencing grade trypsin (Promega, Madison, WI, USA) were added in 1:25 enzyme-to-substrate ratio (w/w) and proteins were digested at 37 °C overnight. Digestion was stopped by the addition of trifluoroacetic acid (TFA) to reach pH ≤ 2, and precipitated SDC was removed by phase-transfer extraction using water-saturated ethyl acetate.^28^ All samples were evaporated to the dryness.

### Tandem Mass Tag Isobaric Labeling

Tandem Mass Tag (TMT) 10-plex (Thermo Fisher Scientific, Waltham, MA, USA) was used as an isobaric labeling reagent for protein quantification. Each TMT label (0.8 mg) was reconstituted in 82 μL of anhydrous acetonitrile (AcN). A 100 μg of peptides reconstituted in 100 μL of 100 mM TEAB was combined with 41 μL of its respective TMT reagent to get a TMT-to-peptide ratio 4:1 (w/w).^29^ The peptides were labeled at 25 °C for 1 h. The TMT labeling was quenched by addition of 8 μL of 5% hydroxylamine and sample incubation at 25 °C for 15 min. Subsequently, equal amounts of all samples were pooled together and desalted using Discovery DSC-18 solid-phase extraction cartridges previously conditioned by methanol and equilibrated using 0.1% TFA in 5% AcN. Peptides were eluted with 0.05% TFA in 50% ACN, evaporated to dryness, and stored at −80 °C in appropriate aliquots for further analysis.

### High-pH Fractionation

Samples were redissolved in mobile phase A (2% AcN/10 mM NH_4_FA), and 372 μg was injected on an UltiMate 3000 RSLC system (Thermo Scientific, Bremen, Germany). Peptides were separated using an XBridge BEH column C18, 2.5 μm, 2.1 μm × 150 mm (Waters, Milford, MA, USA) in a linear gradient of mobile phase B (80% AcN/10 mM NH_4_FA) at a flow rate of 0.3 mL/min. The gradient was running from 0% B to 2% B in 2 min followed by 2% B to 20% B in 9 min, from 20% B to 50% B in 41 min, and from 50% B to 52% B in 5.5 min. In total, the gradient time was 57.5 min. Fractions were collected in a 96-well polypropylene plate (Agilent Technologies, Santa Clara, CA, USA) at 45 s from intervals from 3.5 to 57.5 min yielding 72 fractions in volume of 225 μL. Collected fractions were subsequently pooled into 24 fractions containing 15.5 μg of peptides each (Table S4). Finally, 3 μg of peptide material was evaporated and stored at −80 °C for nLC-MS/MS, and the remaining evaporated 12.5 μg were stored at −80 °C for μLC-MS/MS.

### Nanoflow Liquid Chromatography Coupled to Tandem Mass Spectrometry (nLC-MS/MS)

Each fraction (3 μg) was redissolved in a loading solvent (2% AcN/0.1% TFA), and 0.5 μg was injected into an UltiMate 3000 RSLCnano system (Thermo Scientific, Bremen, Germany). The analytical system consisted of a PepMap100 C18, 3 μm, 100 Å, 75 μm × 20 mm trap column, and PepMap RSLC C18, 2 μm, 100 Å, 75 μm × 250 mm analytical column (both from Thermo Scientific). All fractions were loaded in a trap column at a flow rate of 5 μL/min for 5 min. Peptides were separated in a linear gradient of mobile B (80% AcN/0.1% FA) running from 2% B to 34.5% B in 70 min followed by 34.5% B to 45% B in 10 min at a flow rate of 250 nL/min. Eluted peptides were introduced into Q Exactive Plus mass spectrometer via a Nanospray Flex ion source (both Thermo Scientific, Bremen, Germany). Positive ion full-scan MS spectra were acquired using the parameters specified in Table 1. All fractions were analyzed in two technical replicates.

**Table 1:** LC-MS/MS Settings for All Analytical Approaches

	μLC-MS/MS			nLC-MS/MS		
**method**	1D-TMT	2D-TMT	1D-LFQ	1D-TMT	2D-TMT	1D-LFQ
TopN	10	10	12	10	10	10
MS1 resolution	35 K	35 K	35 K	70 K	70K	70K
MS1 AGC target	3.00 × 10^6^	3.00 × 10^6^	3.00 × 10^6^	3.00 × 10^6^	3.00 × 10^6^	3.00 × 10^6^
MS1 IT	110 ms	110 ms	110 ms	100 ms	100 ms	50 ms
MS2 resolution	35K	35K	17.5K	35K	35K	17.5K
MS2 AGC target	2.00 × 10^5^	2.00 × 10^5^	1.00 × 10^6^	1.00 × 10^5^	1.00 × 10^5^	1.00 × 10^6^
MS2 IT	120 ms	120 ms	120 ms	60 ms	60 ms	120 ms
isol. window	1.6	1.6	2	1.6	1.6	2
NCE	32	32	28	33	33	28
min. AGC target	1.0 × 10^3^	1.0 × 10^3^	1.0 × 10^3^	1.0 × 10^3^	1.0 × 10^3^	1.2 × 10^4^
charge	≥2 to ≤ 8					
DE	7 s	7 s	7 s	17 s	17 s	17 s
method length	275 min	107 min	275 min	275 min	107 min	275 min
gradient length	250 min	83 min	250 min	240 min	80 min	240 min

### Microflow Liquid Chromatography Coupled to Tandem Mass Spectrometry (μLC-MS/MS)

Peptide separation was processed after injection into an UltiMate 3000 binary RSLC system (Thermo Scientific, Bremen, Germany) configured as previously described.^3^ In brief, each fraction (12.5 μg) was redissolved in mobile phase A (3% DMSO/0.4% acetic acid (HAc/0.1% FA) and 10 μg of the sample was injected into a HALO Peptide ES-C18, 2.7 μm, 160 Å, 1.0 mm × 250 mm analytical column (Advanced Materials Technology, Wilmington, DE, USA). Peptides were separated in a linear gradient of mobile phase B (78% AcN/3% DMSO/0.4% HAc/0.1% FA) running from 0.2% B to 2% B in 4 min followed by 2% B to 30% B in 70 min and by 30% B to 45% B in 9 min at a flow rate of 68 μL/min. Eluted peptides were introduced into a Q Exactive Plus mass spectrometer via an EASY-Spray ion source equipped with an HESI-II probe (Thermo Scientific, Bremen, Germany). The settings of MS and MS/MS acquisition were optimized to reach the utmost protein coverage. Optimized parameters for the full-scan MS method are listed in Table 1. All fractions were analyzed in one technical replicate.

### Data Processing

Survey MS and MS/MS spectra were processed by MaxQuant^30^ v1.6.14.0 with its built-in search engine, Andromeda.^31^ MS/MS spectra were searched against the *Oryctolagus cuniculus* reference proteome (UP000001811) downloaded from Uniprot in April 2021. Contaminants were identified according to the MaxQuant-implemented database. For TMT data processing, reporter ion in MS2 was selected as a group-specific parameter. Reporter ion intensities were corrected by TMT correction factors provided by the manufacturer. Reporter mass tolerance was set to 3.16 mDa. Unique and razor peptides were selected for quantification with a minimum ratio count of 2. Precursor tolerance for the first search was set to 20 ppm, for the second search from recalibrated spectra to 4.5 ppm (with individual mass error filtering enabled). Only peptides with maximal charge *per* peptide *z* = 7, minimal length 7 amino acids, and maximal mass of 4600 Da were considered to analysis. Alkylation of cysteine sulfhydryl groups to dithiomethane was set as fixed modification. Protein N-term acetylation and methionine and proline oxidation were set as variable modifications. Trypsin/P with maximum 2 missed cleavages was specified as a protease. For modified peptides, the minimal Andromeda score was 40 and minimal delta score was 6, respectively. Peptide spectrum matches (PSM) were filtered using a false discovery rate of 0.01 employing a target-decoy approach using reversed protein sequences. Also, PSM were filtered by their precursor intensity fraction (PIF) to value ≥ 0.75. The Remaining MaxQuant parameters have been kept in their default value.

### Data Treatment and Evaluation

Output files from MaxQuant were processed using Perseus 1.6.14.0.^32^ Potential contaminants and proteins identified by site and by reverse sequence were filtered out. Corrected reporter ion intensities were converted to log_2_ values, and only proteins with valid values in each TMT channel in at least one technical replicate (nLC) were considered for further analysis. Reporter intensities were subsequently normalized by subtraction of median from each observed value in each TMT channel. Redundant hits of proteins were searched and filtered out based on the number of razor and unique peptides as follows: 1) 0 unique peptides but leading razor peptides > protein included other proteins filtered out; 2) leading razor and unique peptides > protein included and other proteins filtered out; 3) proteins with the same fasta names but different gene names > checked according to appropriate peptide sequences > filtering according to razor and unique peptides; 4) filtered out peptides with 0 unique proteins and 1 razor peptides, if there was alternative. Uncharacterized proteins were annotated using String db^33^ based on gene names, if present. Finally, protein levels in DAU-treated samples were compared with controls by moderated *t*-tests based on the Linear Models for Microarray Data (LIMMA) approach^34,35^ in R 4.0.5.^36^ Significantly changed proteins were annotated by Gene Ontology (GO) terms: the biological process, cellular component, and molecular function, and 1D enrichment for these GO terms with Benjamini–Hochberg correction (FDR 0.05) was performed in Perseus.^37^

## Results

### Establishment of Oroteolysis and Peptide Labeling Workflow

**Figure 1:**
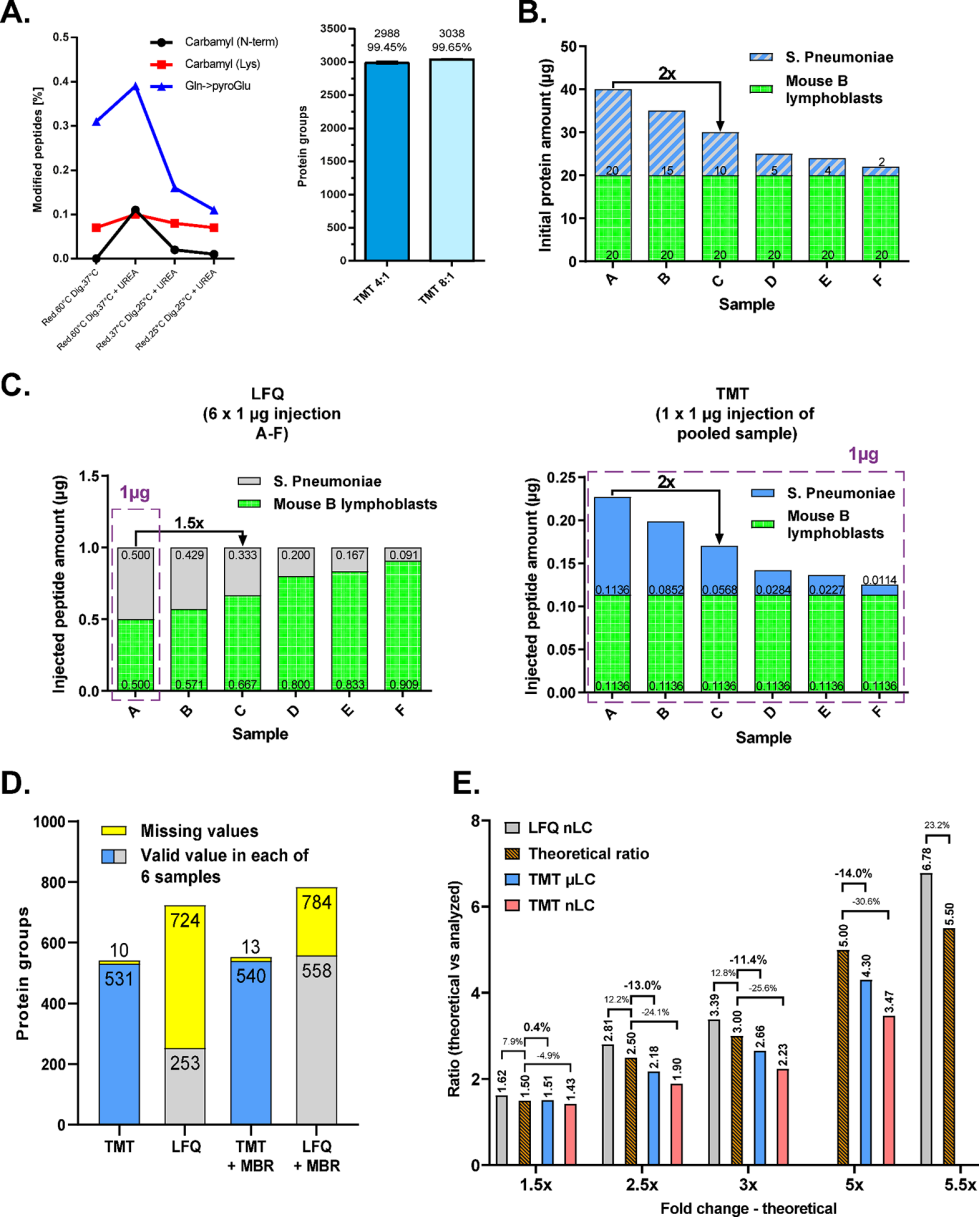
Comparison of sample preparation and quantification strategy**.** (A) Percentages of peptides that were assigned to contain N-terminal carbamylation (black), lysine carbamylation (red), and pyroglutamic acid (blue). The samples were prepared with or without added urea under two different temperatures of disulfide bond reduction (60 °C vs 37 °C) and digestion (37 °C vs 25 °C). The bar plots illustrate the ratio (%) of TMT labeling efficiency for the two TMT to protein ratios (4:1 vs 8:1) with the total number of quantified proteins in the sample. The error bars represent the standard deviation (*n* = 3 replicates). (B) Accuracy of TMT-based and LFQ quantification was compared using *S. pneumoniae* (strain Rx1) proteins spiked into the protein matrix of mouse B lymphoblasts at six ratios. (C) Quantity of each proteome and its ratios for the samples from Figure 1B were injected sequentially in the LFQ approach and single injection of a multiplexed sample in the TMT approach. (D) Bar plots illustrating the number of quantified proteins and instances of missing values for TMT-based and LFQ quantification with and without the incorporation of the match-between-runs algorithm (MBR) within MaxQuant. (E) Comparison of the theoretical and analyzed ratios for TMT-based and LFQ quantification using either μLC or nLC. The discrepancy (%) between the theoretical and analyzed ratios for each technique is presented above the bars.

Heat-induced disulfide bridge reduction and protein digestion in urea-containing buffers are associated with an increased risk of the occurrence of artificial peptide modifications. Therefore, the temperature was a critical condition given the presence of urea in the lysis buffer.^38^ We performed basic optimization of the temperature used for reduction of disulfide bonds and digestion (Supporting Methods, Table S1). The temperature of 37 °C was selected for the disulfide reduction due to the low number of artificially modified peptides (Figure 1A). A further decrease in temperature for disulfide reduction to 25 °C did not significantly decrease the number of modified peptides in our experimental settings (Figure 1A) and led to a decrease in the number of identified proteins (data not shown). As proteins were acetone-precipitated prior to the digestion, the same temperature (37 °C) could be employed for the digestion step as well as because potential issues related to urea were mitigated. Being a critical step for protein quantification, the labeling efficiency was assessed by searching the achieved data with TMT labels set as variable modifications. The TMT labeling efficiency was assessed as a ratio (%) of N-terminally labeled peptides (Nt_i_) along with labeled lysins (Nk_i_) to total number of N-termini (Nt_t_) and lysins (N*k*_t_).^39^
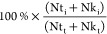


Both reduced (4:1) and recommended (8:1) TMT-to-peptide ratio was tested as described in Supporting Methods and both approaches achieved labeling efficiency of 99.5% (Figure 1A), if the labeling procedure was performed in 100 mM TEAB. Reduced concentration of TMT (4:1) resulted in 2988 quantified proteins means only 1.65% difference in favor of the higher employed ratio (8:1, 3038 proteins). Based on these pilot experiments, 100 mM TEAB and a TMT-to-peptide ratio of 4:1 were applied in further experiments.

### Comparison of TMT Peptide Labeling and Label-Free Approach

The yield and accuracy of TMT labeling (TMT) and label-free (LFQ) quantification were compared using a *S. pneumoniae* (strain Rx1) proteins spiked into the protein matrix of mouse B lymphoblast at six ratios (Supporting Methods and Figure 1B) and analyzed in three technical triplicates (Figure 1C). The TMT multiplexing approach consumed only ≈16 h of instrument time, whereas LFQ analysis required ≈93 h to finish the whole experiment. In total, higher number of protein groups was identified in LFQ, but also along with higher number of missing values subsequently mitigated using Match-between-runs algorithm (MBR) integrated in MaxQuant. By contrast, the number of missing values in the TMT experiment was very low regardless of the application of MBR (Figure 1D). Protein ratios estimated by both TMT and LFQ analysis were distinct from the expected values. A combination of TMT approach and nLC-MS/MS strongly underestimated the theoretical ratios toward more abundant protein levels, whereas the reverse trend was observed in LFQ. However, the compression of TMT-MS2 ratios was less noticeable in μLC-MS/MS data compared to nLC-MS/MS. The experimentally achieved TMT-MS2 ratios were significantly closer to the theoretical (expected) values especially toward lower concentration, for instance, Δ 0.4% versus −4.9% at a 1.5 fold-change or Δ −11.4% versus −25.6% at a 3 fold-change) (Figure 1E). The approach to increase the number of quantified proteins was also tested by comparing 1D with simple 2D LC-MS/MS (8 fractions) with more extensive 2D LC-MS/MS (24 fractions) (Table S4 and Figure S1A,S1B). The initial 1D nLC-MS/MS analysis of the mouse B lymphoblasts lysate digest (Supporting Methods) served as the benchmark for quantification, resulting in the identification of 1632 proteins. In comparison, the simple 2D nLC-MS/MS analysis with previous high-pH fractionation (8 fractions) led to the quantification of 4517 proteins. The number of quantified proteins was further increased by modifying the fractionation protocol to obtain the final 24 concatenated fractions. The resulting analysis yielded the quantification of 6456 proteins (Figure S1A). Only a small number of proteins were unique to either the 1D or 2D (8 fractions) approach. Therefore, we opted to employ a more comprehensive 24-fraction approach, which was capable of quantifying the highest number of proteins while also generating 1938 unique proteins (Figure S1). The further mapping of proteome changes induced by DAU was conducted via LC-MS/MS analysis of concatenated 24 fractions.

### Animal Data

The characteristics describing cardiac status in each rabbit included in the study (DAU1–3 and CTRL1–3) at the end of the study are summarized in Table 2. Altogether, the data from DAU-treated animals included in this study showed the development of severe cardiotoxicity characterized by profound decline of the left ventricular (LV) systolic function (LV fractional shortening and LV ejection fraction examined by echocardiography) from the initial values and marked rise of plasma biomarker of cardiac damage (cTnT). Furthermore, congestion in lungs (increased lung weight normalized on the body weight and hydrothorax) was observed in all DAU-treated animal as a consequence of the LV dysfunction along with increased heart weight normalized on the body weight and macroscopic changes to the heart (mainly LV dilation). One DAU-treated animal (DAU3) suffered by end-stage heart failure and died spontaneously before invasive examination of LV function at the end of experiment. In this case, the necropsy was performed immediately, so the myocardium sample was taken for further analysis in the standard way as in other animals. No changes in comparison with the baseline were found in the control group.

**Table 2:** Clinical Characteristics of Rabbits with Severe Experimentally Induced Anthracycline Cardiotoxicity (DAU1, DAU2, and DAU3) and Corresponding Controls (CTRL1, CTRL2, and CTRL3)^a^

			DAU 1	DAU 2	DAU 3	CTRL 1	CTRL 2	CTRL 3	**mean DAU/mean CTRL**
Complete survival			Y	Y	N*	Y	Y	Y	NA
ECHO LV systolic function	LVFS (%)	initial	43.2	44.3	42.9	43.6	42.9	42.3	1.01
		end	24.4	25.9	16.8	42.4	42.8	42.9	0.52
		ΔLVFS	18.8	19.2	26.1	1.2	0.1	–0.6	NA
	LVEF (%)	initial	77.8	79,0	77.3	78.4	77.3	76.6	1.01
		end	51.9	53.0	37.7	77.1	76.5	77.0	0.62
		ΔLVEF	25.9	26.0	39.6	1.3	0.8	–0.4	NA
LV catheterization index d*P*/d*t*_max_ (mmHg/s)			3773	2874	NA*	8459	8811	7221	0.41c
cardiac troponin *T* (cTnT) in plasma (μg/L)		initial	0.004	0.004	<0.003	0.003	0.003	0.005	1.00
		end	0.167	0.238	0.177	0.006	0.008	0.012	20.08
heart weight/BW (g/kg)			3.17	4.00	3.27	2.18	2.60	2.77	1.38
lung weight/BW (g/kg)			6.54	5.28	5.41	2.71	3.01	2.89	2.00
hydrothorax (mL)			2.6	18.5	92.0	0	0	0	NA

a Complete survival –survival until the scheduled end of the experiment (*the animal died before LV catheterization, i.e., right after echocardiographic examination, due to end-stage heart failure). Noninvasive echocardiographic (ECHO) examination of LV systolic function was performed. Left ventricular fractional shortening (LVFS) and LV ejection fraction (LVEF) at the beginning (initial) and at the end of experiment (end) are shown together with the difference of the values obtained at the beginning and at the end of study (ΔLVFS and ΔLVEF). LV catheterization, invasive examination of the left ventricular function performed at the end of experiment: d*P*/d*t*_max_, maximum pressure raise in the isovolumic phase of systole (an index of systolic function). *Cardiac troponinT* (cTnT) –plasma concentrations of cardiac troponin T determined by a high-sensitive assay as a biomarker of cardiac damage. Heart weight (wet) found during necropsy normalized on the body weight (BW) as a biomarker of trophic changes to the heart, lung weight (wet) normalized on the body weight (BW), and hydrothorax (pleural effusion) as signs of blood congestion found during necropsy. Y, yes; N, no; N/A, not available.

### Histological Analysis

**Figure 2:**
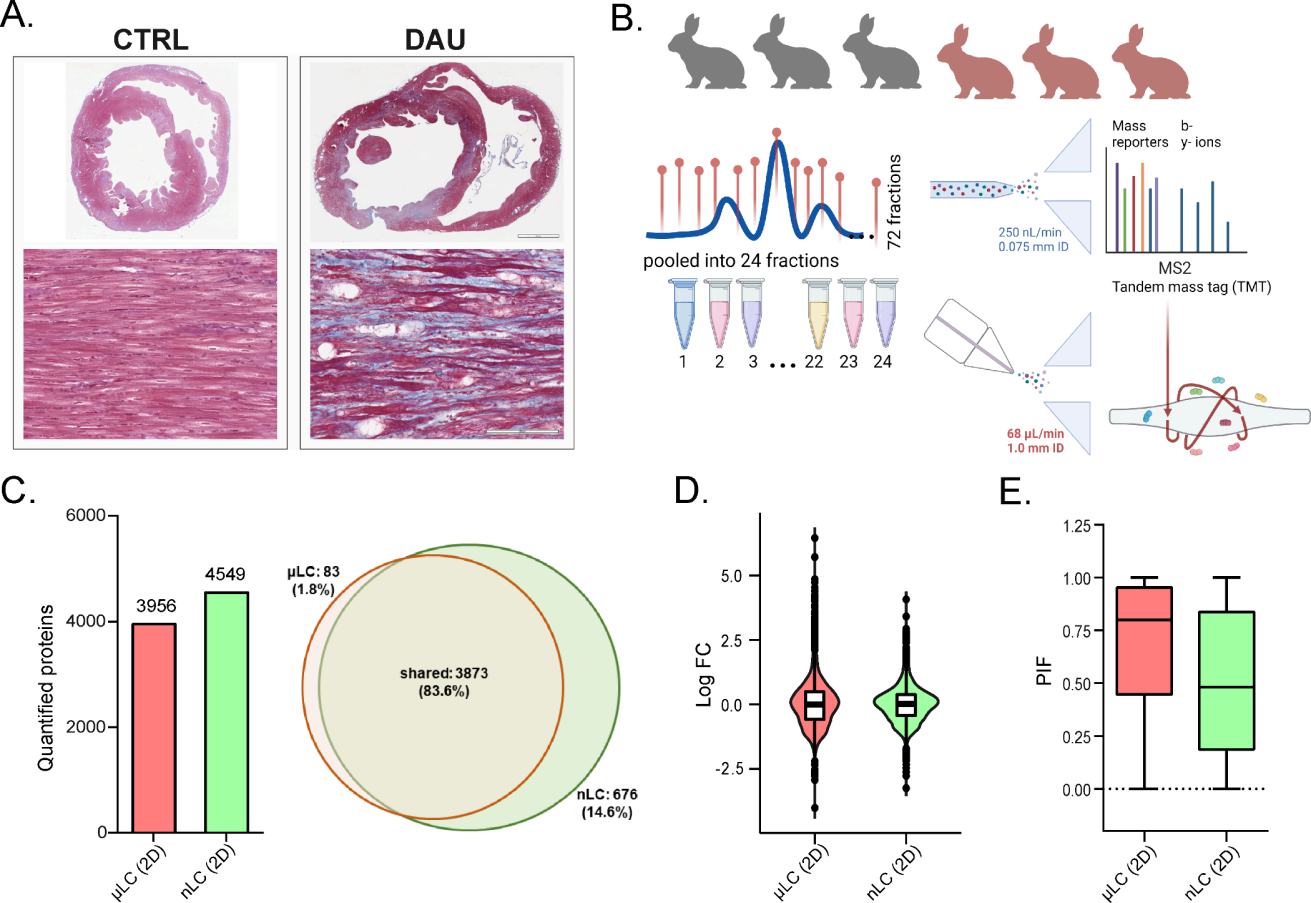
Morphological changes in the rabbit heart, schematic workflow and results of the comparison of μLC vs. nLC techniques utilized for wide-scale analysis of myocardial proteome changes. (A) Transverse section of a normal control (CTRL) heart compared with a heart suffering from advanced daunorubicin (DAU)-induced cardiomyopathy. In the DAU group, there is macroscopically seen significant dilatation of both ventricles. In the microscopic picture of the left ventricular myocardium, degenerative changes (myofibril disintegration to vacuolization of the cytoplasm and disintegration of the cell nucleus) predominate, leading to the death of groups of cardiomyocytes. The remnants of decayed cardiomyocytes are removed by macrophages (in the presence of mild mononuclear infiltrate), and they are replaced by connective tissue (blue color) forming a marked myocardial scarring. (B) Schematic workflow overview of the wide-scale analysis of myocardial proteome changes induced by severe ANT cardiomyopathy. (C) Number of quantified proteins in TMT-labeled and multiplexed myocardium samples determined using either μLC or nLC, following the previous high-pH fractionation. The Venn diagram illustrates the proportion of unique and overlapped proteins quantified by μLC (orange) and nLC (green) in myocardium samples after previous high-pH fractionation. (D) Violin plots representing log_2_ fold-change between DAU-induced and control groups demonstrate a superior dynamic range for μLC (orange) in comparison to nLC (green). (E) Enhanced resolution of TMT ions in MS2 was substantiated by box plots of PIF (parent ion fraction) values for all PSMs identified by μLC (orange) and nLC (green).

Histological analysis revealed extensive morphological damage to the myocardium of DAU-treated animals (Figure 2A), particularly in the LV and interventricular septum. These changes comprised mainly distinct focal degenerative changes to cardiomyocytes characterized by loss of myofibrils and vacuolization of the cytoplasm. Many cardiomyocytes showed advanced degenerative changes resulting in cell death. The healing process, i.e., replacement fibrosis accompanied by a mild mononuclear infiltrate (including macrophages removing decayed cells), resulted in the formation of fibrous scars of variable extent. At the macroscopic level, LV or biventricular dilation (in end-stage heart failure) was observed. In contrast, normal morphology of the myocardium was observed at both microscopic and macroscopic levels in the control group.

### Wide-Scale Analysis of Myocardial Proteome Changes Induced by Severe ANT Cardiotoxicity

Based on method and system optimization described above, 2D-TMT μLC-MS/MS (μLC) analysis of protein level changes in rabbit myocardium related to severe DAU-induced cardiomyopathy (Figure 2A) was performed, and the results were compared to 2D-TMT nLC-MS/MS equivalent (nLC) (Figure 2B). Globally, 4729 items were attained across both data sets after filtering of protein inference redundancy.^40^ In total, 3956 proteins were quantified with valid values for all TMT channels in μLC data set and 4549 proteins were available with at least one valid value from two technical replicates (4489 in first replicate and 4458 in second replicate, respectively) in each TMT channel in nLC. Across both data sets, 83.6% of quantified proteins were shared, whereas 83 proteins were quantified uniquely in μLC and 676 proteins were unique for nLC (Figure 2C). However, μLC-MS/MS exhibited a broader (log_2_) fold-change protein distribution (Figure 2D), better resolution of TMT ions in MS^2^ (Figure 2E) consistent with the low compression of TMT ratios in μLC (Figure 1E), higher probability of PSM (Figure S2B), and narrower peak width (Figure S2C).

**Figure 3:**
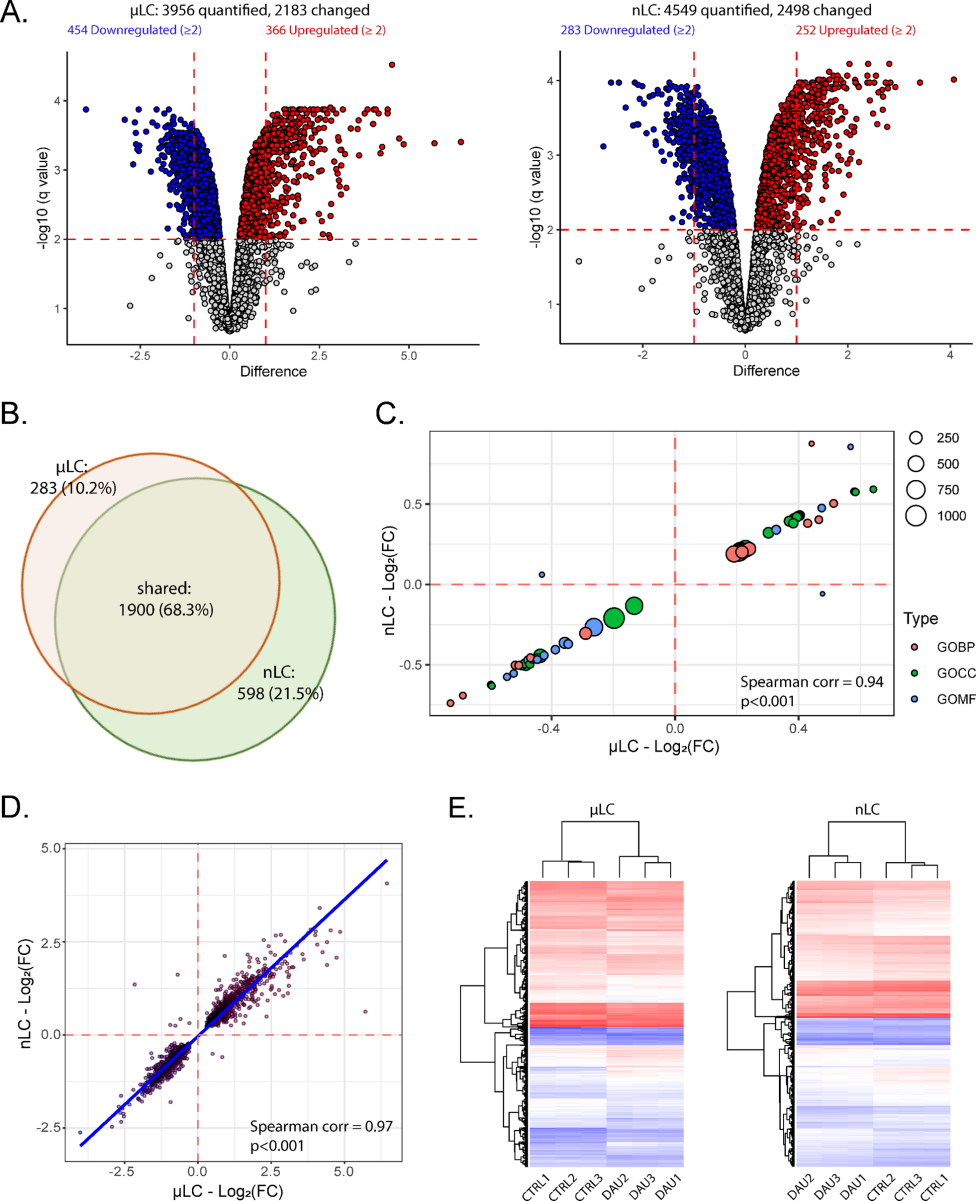
Concordance rate between two data sets representing μLC or nLC techniques utilized for wide-scale analysis of myocardial proteome changes was evaluated. (A) Volcano plots demonstrating that both μLC and nLC techniques confirmed substantial DAU-induced proteome dysregulation. Significant proteins (Benjamini-Hochberg FDR < 1%, illustrated by a horizontal red line) are indicated by blue (downregulated) and red (upregulated) colors. The μLC technique was capable of quantifying a smaller number of proteins (3956 vs 4549) but demonstrated a higher percentage of significantly changed proteins (down- and upregulated) with a fold-change ≥ 2 (illustrated by vertical red lines) in comparison to the nLC technique (21% vs 12%). (B) Venn diagram depicting the number and overlap of significantly changed proteins quantified by μLC (orange) and nLC (green). (C) Scatter plot representing 2D enrichment analysis (Spearman correlation coefficient = 0.94, *p* < 0.001), (D) Scatter plot depicts correlation of significant protein changes determined by μLC and nLC (Spearman correlation coefficient = 0.97, *p* < 0.001). (E) Heat maps exhibiting the differentially expressed proteins between the DAU-treated and control groups, as analyzed by either μLC or nLC. Data were log_2_ transformed and normalized by subtraction of median from each observed value prior to clustering analysis.

From all quantified proteins, levels of approximately 55% proteins were found significantly changed due to the induction of severe anthracycline cardiomyopathy. In particular, 2183 (55.2%) and 2498 (54.9%) significantly changed proteins were quantified in μLC and nLC, respectively (Figure 3A and Supplementary Table 1). From those, 1900 were shared by both data sets, 283 changed proteins were quantified uniquely in μLC and 598 changed proteins were unique for nLC (Figure 3B). Hence, nLC performed moderately better herein, as it detected more significantly changed proteins. On the other hand, when looking for significantly changed proteins with a higher fold-change (arbitrary selected fold-change ≥ 2), than TMT μLC-MS/MS identified 21% (820) proteins, while TMT nLC-MS/MS only 12% (535) proteins (Figure 3A). Most of the significantly changed proteins with a fold-change of ≥2, which were detected using the μLC analysis but not nLC analysis, were upregulated due to the severe cardiotoxicity development. In addition, a high correlation of GO terms designed based on 2D enrichment analysis (Spearman corr. coefficient = 0.94, *p* < 0.001) (Figure 3C) as well as high correlation of significant protein changes determined by μLC and nLC was affirmed (Spearman corr. coefficient = 0.97, *p* < 0.001) (Figure 3D). Both data sets confirmed the consistent shift of myocardial proteome due to induction of severe DAU cardiomyopathy from controls in all 3 biological replicates (Figure 3E).

### Changes of Myocardial Proteins Induced by Severe Daunorubicin Cardiomyopathy

**Figure 4:**
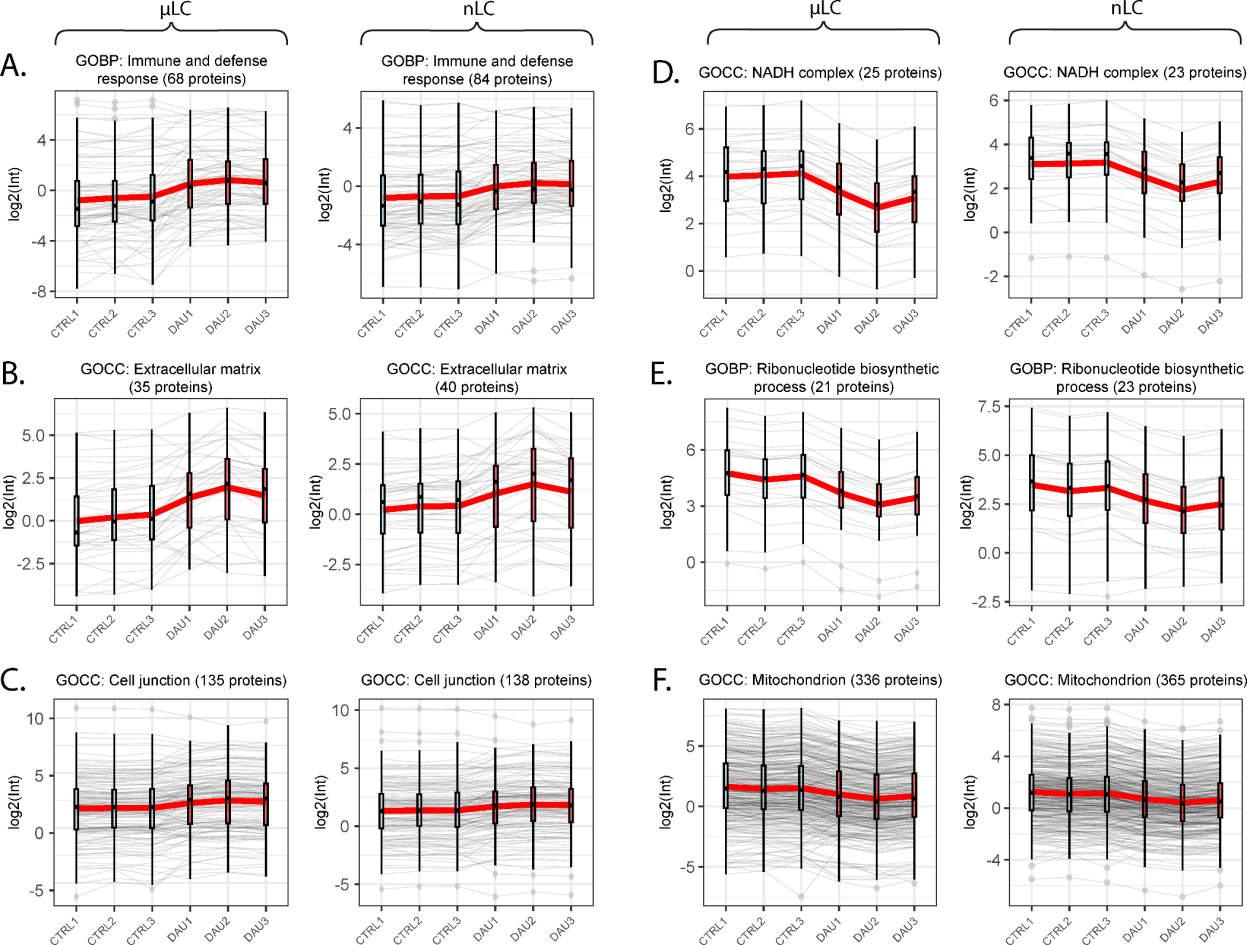
The figure exhibits the profile plots of protein level changes in Gene Ontology (GO) clusters, quantified by either μLC or nLC techniques. The clusters that exhibited a significant upregulation in the DAU group vs control group are represented by (A) GOBP–immune and defense response, (B) GOCC–extracellular matrix, and (C) GOCC–cell junction. The clusters that exhibited a significant downregulation are associated with (D) GOCC–NADH complex, (E) GOBP–ribonucleotide biosynthetic process, and (F) GOCC–mitochondrion. The mean value of the logarithms of the intensities within each cluster is indicated by a red thick line.

Data obtained by both μLC and nLC revealed significant upregulation of many proteins related to immune and defense response (68 vs 84 proteins; μLC vs nLC) (Figure 4A), extracellular matrix (35 vs 40 proteins) (Figure 4B), and cell junction (135 vs 138 proteins) (Figure 4C). Among the proteins of the immune and defense response GOBP cluster (Figure 4A), interferon response proteins like the ISG15 ubiquitin-like modifier (ISG15), protein with tetratricopeptide repeats 2 and 3 (IFIT and IFIT3), 2–5 oligoadenylate synthase (OAS2 and OAS3), and STAT1 or dynamin-type G domain-containing protein (MX2) were strongly upregulated in both μLC and nLC analysis. Noteworthy, ISG15 was also the most upregulated protein due to severe DAU cardiomyopathy in both data sets. Furthermore, pentaxin (CRP), interleukin-18 (IL18), complement comment proteins (C1S, C3, C6, C8A, C8B, and C8G) and lipopolysaccharide-binding protein (LBP) were other important inflammatory proteins found changed by both μLC and nLC from the same GOBP cluster.

Regarding GOCC cluster, extracellular matrix proteins (Figure 4B), important changes toward upregulations were found both using μLC and nLC analysis in proteins: (a) regulating extracellular matrix remodeling (cellular communication network factor 2–CTGF, latent transforming growth factor beta binding protein 2–LTBP2), (b) matricellular proteins (tenascin C–TNC, periostin–POSTN, and osteonectin–SPARC), (c) glycoproteins (fibulin 2–FBLN2, SMB domain-containing protein/vitronectin - VTN), (d) proteoglycans (asporin–ASPN, biglycan–BGN, and decorin–DCN), (e) collagenous proteins (collagen type XII alpha-1 chain – COL12A1 and collagen type VI alpha 3 chain–COL6A3) and (e) galectin 3 (LGALS1) and related galectin-3-binding protein (LGALS3).

The cell junction GOCC cluster showed a marked but diverse response to induction of the severe anthracycline cardiotoxicity. Cadherins, the transmembrane glycoproteins forming cell–cell connections, were found to decrease using both LC-MS analyses. This concern cadherin 2 (N-cadherin, CDH2), which is the main cadherin of cardiomyocytes located in their intercalated discs. Other important proteins related to the intercalated disc were decreased, e.g., desmoplakin (DSP), which provides the link from the desmosomes to the intracellular intermediate filaments or alpha and beta catenins that mediate interaction of N-cadherin with the intracellular F-actin cytoskeleton. Furthermore, the main gap junction protein of the intercalated disc (Gap junction alpha-1 protein/connexin 43–GJA1) was also decreased. Besides that, cadherin 5 (VE cadherin, CDH5) involved in the adhesion of the vascular endothelial cells was also decreased due to the treatment. Integrins, the transmembrane heterodimeric receptors important for cell adhesion to the extracellular matrix, were mostly upregulated (ITGAV, ITGA7, ITGB1, ITGB2, and ITGB5) as determined by both μLC and nLC analysis, but some of the alpha subunits (ITGA5, ITGA6, and ITGA9) were found downregulated. Talin, which interacts with the intracellular domain of the integrins, showed an isoform-specific response to severe anthracycline cardiotoxicity as determined by both μLC and nLC analysis (talin 1–TLN1 was upregulated, while the opposite was true for talin 2 – TLN2). Proteins of the dystroglycan complex (dystrophin – DMD and dystrobrevin–DTNA) were decreased in the DAU-treated hearts. In contrast, both μLC and nLC analysis found a marked increase in intermediate filament desmin (DES), which anchor myofibrils to the costamere and desmosomes, and the same was true for noncardiomyocyte intermediate filament vimentin (VIM). Key proteins of normal cardiac sarcomere such as myosin 6 (MYH6), myosin 7 (MYH7), actin alpha cardiac muscle (ACTC1) and titin (TTN) were concordantly found markedly decreased in severe anthracycline cardiotoxicity.

High concordance was also attained between μLC and nLC results in the clusters showing clear downregulation of myocardial proteins due to cardiotoxicity induction, e.g., proteins related to the NADH dehydrogenase complex (i.e., subunits of complex I of the respiratory chain) were severely decreased due to the DAU treatment as determined by both μLC and nLC (25 vs 23 proteins; e.g., NDUFA6, NDUFB3, NDUFB6, and MT-ND1) (Figure 4D), and similar was true for proteins of the ribonucleotide biosynthetic process (21 vs 23 proteins) (Figure 4E). In the latter cluster, vast majority of proteins was related to ATP synthesis, and approximately half of these proteins were subunit of complex V of respiratory chain (e.g., ATP5B, ATP5F1, ATP5I, and MT-ATP6). In the light of this data, it is not surprising that mitochondrial proteins ranked among those most altered in the severe anthracycline cardiotoxicity in both μLC and nLC analysis (336 vs 365 proteins) (Figure 4F). Vast majority of these proteins (≈85%) showed a decrease in abundance, and the most profound changes in both analyses were related to glutamic-pyruvic transaminase 2 (GPT2), glycine C-acetyltransferase (GCAT), and glycine *N*-acyltransferase (GLYAT). Less common was an increase in mitochondrial proteins due to the cardiotoxicity which was observed, e.g., in BCL2-associated X (BAX) or calcium-binding mitochondrial carrier protein SCaMC-1 (SLC25A24).

Both μLC and nLC described the induction of the expression of natriuretic peptides (ANP and BNP) in the myocardium with severe anthracycline cardiotoxicity, which is a hallmark of LV dysfunction. Both μLC and nLC also identified decreased abundance of the cardiac isoform of troponin I (TNNI3) and corresponding TNNI3 interacting kinase (TNNI3K) in the severe anthracycline cardiotoxicity, while the slow skeletal muscle isoform of troponin I (TNNI1) was increased. Besides that, multiple signaling proteins that are typically present in lower abundance were successfully identified altered by both μLC and nLC (e.g., SIRT3, SIRT 5, CAMK2G, CAMK2D, PRKCD, PDK1, PDK2, PDK3, RRM2B, and PRKACB or multiple subunits of AMPK like PRKAA2, PRKAB2, and PRKAG2).

## Discussion

nLC system is tightly connected with modern MS-based proteomic analysis of complex biological samples, but recent high-impacted publications pointed out the alternative in the form of μLC, LC-MS/MS operating in the flow rates above 50 μL/min.^3,4,41,42^ For the last three years, a number of nLC studies exploiting distinct quantification approaches with the aim to determine protein levels in myocardial tissues have been published. In total, 3136 of proteins in a porcine myocardium were identified and successfully quantified using a standard PepMap nLC-column (C18, 75 μm × 500 mm, 175 min-gradient) and a Q Exactive HF-X.^43^ A similar nLC study reached 2329 proteins from 34 samples of porcine myocardial tissues using an Orbitrap Elite,^23^ and 2164 proteins from 29 FFPE samples of human myocardium were reached in 2D nLC analysis on a Q Exactive HF-X.^24^ In 2021, 1655 proteins of mice myocardium were quantified using 2D-TMT nLC on an Orbitrap Fusion.^44^

Building on our previous work,^3^ we optimized our high-flow LC system hyphenated to a Q Exactive Plus (Table 1) to compete with the traditional nLC system on the same MS platform and to efficiently describe the proteome of the DAU-induced cardiotoxicity in the rabbit myocardium. The urea-containing buffer, previously shown to be effective for protein extraction from homogenized myocardium tissues,^11^ was employed here with adjustments of the reduction conditions (37 °C) to minimize artificial pyroglutamate formation and carbamylation (Figure 1A).^45^ When a temperature of 37 °C was used for the reduction step in a urea-containing buffer, carbamylation levels remained very low (≤0.1%), comparable to those in the standard protocol without urea. A further decrease in the temperature during disulfide bond reduction did not result in any significant improvement in minimizing artificial modifications but instead led to a decrease in overall protein identification, likely due to ineffective disulfide bond reduction under the given experimental condition. Due to the achievement of satisfactory results, further optimization of the extraction or digestion protocol were deemed unnecessary. Isobaric labeling of peptides, as one of the most popular proteomic approaches,^46^ offers high multiplexing capability, improving throughput, reproducibility, sensitivity, and instrument time efficiency. On the other hand, the financial cost of TMT tags could be a limitation, especially in the case of a high number of samples. In addition, coisolation and cofragmentation of TMT-labeled peptides with similar *m*/*z* may lead to ratio distortion and negatively affect the accuracy of the protein quantification.^47^ In 2019, Zecha et al. demonstrated nearly complete (>99%) in-solution TMT labeling using 1/8 of a TMT labeling reagent with excellent reproducibility.^29^ In our study, 1/2 of TMTs achieved comparable protein coverage (Figure 1A), and nearly complete peptide labeling (>99%, Figure 1A). The instrument time required for the TMT multiplex analysis was significantly lower (16 h) in comparison with the instrument time for the LFQ approach (96 h) (Figure 1C). In addition, the TMT approach was superior to the LFQ in the accuracy of peptide identification as the LFQ experiment was complicated by the missing data. Although the match-between-runs method (MBR) increased valid values, the number of quantified proteins was comparable for TMT without MBR to LFQ supported by MBR, which can be associated with incorrect transfer of an identification (Figure 1D).^48^ Ratio distortion in TMT-MS2, often reported in the literature,^49−52^ was observed in our nLC-based data, particularly for lower-abundance proteins in a complex matrix. However, LFQ overestimated protein quantities (Figure 1E), whereas μLC-based TMT-MS2 ratios were more accurate than those obtained from nLC, especially for low-abundance-labeled peptides (Figure 1E). More accurate quantification using μLC-based TMT-MS2 analysis of complex samples builds on previously published data^2,38,41,42^ as well as our findings regarding PIF (coisolation interference, Figure 2E) and reduced peak widths (Figure S2C), which is a result of increased peak capacity. To the best of our knowledge, this is the first demonstration of utilizing reduced coisolation interference and narrower peak widths to improve the accuracy of TMT-based quantification. Highlighting this point is particularly important in a multidisciplinary journal with an interdisciplinary audience, as it facilitates a deeper understanding of the issue of ratio distortion and its significant impact on protein fold-change analyses using nLC-MS/MS, a challenge often overlooked or insufficiently described in such contexts. These findings prompted us to prioritize the TMT multiplexing strategy for comprehensive mapping of proteome changes associated with cardiotoxicity development with a focus on comparing nLC and μLC workflows.

Two-dimensional liquid chromatography (2D LC) became a key part of exploratory bottom-up proteomics, providing a complex protein mixture ineffectively separable in simple one-dimension (1D). However, a current trend is data acquisition in one LC-MS/MS run instead of analysis extended by an additional LC dimension.^42^ To maximize the number of quantified proteins in myocardium samples, we decided to compare the recovery of both 1D and 2D LC prior MS considering the limited scan rate and resolution of Q Exactive Plus in comparison to next-generation orbitrap analyzers that followed (HF-X, Exploris 480, Elite, etc.). According to the expectations, 1D and 2D LC-MS/MS (8 fractions) (Table S3) were strongly outperformed by extensive 2D LC-MS/MS (24 fractions) (Table S4 and Figures S1A,S1B). The transfer of the methods from 1D to 2D was accompanied by the optimization of the maximum injection time in MS2 and other acquisition parameters (Table 1), resulting in a similar identification rate for 2D-μLC-MS/MS (12.1% PSMs per submitted MS/MS scans) compared to 2D-nanoLC-MS/MS (12.7%). Final mapping of proteome changes induced by DAU was therefore realized as 2D μLC- and nLC-MS/MS analysis of concatenated 24 fractions. We presumed that extensive fractionation may better manage the challenging dynamic range of protein expressed in the myocardium under normal and pathological circumstances and thus help to cover also the low abundance proteins with important regulatory function.^53^

Importantly, the vast majority of quantified proteins was found altered in both LC-MS/MS setups (83.6%), and both confirmed massive DAU-induced proteome dysregulation with exquisite correlation based on determined protein fold-changes (Figure 3). The nLC outperformed μLC setup with 13% increase in hits (10% in each of nLC technical replicates). However, μLC clearly confirmed the massive shift of more than 2000 myocardial proteins induced by DAU (Figure 3E). Probably due to increased signal intensity, the performance of nLC in detection of proteins, including those with significant changes, was slightly better than that of μLC. However, the increased signal intensity comes with trade-offs, such as stability (Figure S3), broader chromatographic peaks (Figure S2) and thus greater susceptibility to sample complexity and potentially increased coelution, which can reduce the overall confidence of peptide quantitation. When focusing on proteins with a higher fold-change (≥2), μLC identified 21% (820) proteins while nLC identified only 12% (535) proteins (Figure 3A). For this purpose, we selected 2-fold-change cutoff as an accepted threshold in proteomic studies.^54^ However, we acknowledge that relying solely on a fold-change threshold may not fully capture differences arising from varying data set distributions, and therefore, it was used only in the conjunction with the statistical significance.

In comparison with the published results reporting the highest number of quantified myocardium proteins using the same number of biological replicates and TMT labeling,^26^ we successfully quantified by 11% more proteins in our 2D nLC analysis and only by 3.5% less in μLC. However, our main aim was to assess the ability of μLC to describe the complex proteomic signature of severe chronic anthracycline cardiotoxicity. The severe phenotype was purposely employed as it was expectable to provide the most profound and diverse myocardial proteome alterations due to the massive morphological changes in all cell types of the myocardium as well as due to the extracellular matrix remodeling together with marked immune and stress response. Histological analysis confirmed that the DAU-treated samples analyzed in this study fulfill these criteria. Furthermore, the myocardial proteome in these DAU was affected by severe heart failure phenotype as our rabbits suffered from marked systolic dysfunction with lung congestion, and one DAU-treated sample (DAU3) represented the end-stage heart failure phenotype.

Altogether with nLC, μLC confirmed the significant upregulation of pathways related to the anthracycline-induced cardiotoxicity phenotype like immune defense response and pointed on coordinated involvement of type I interferon (IFN-I) signaling, which has been recently proposed to be crucial for anthracycline cardiotoxicity development.^55^ In this light, it is interesting to note that the ISG15 ubiquitin-like modifier (ISG15), which is triggered by type I interferon (IFN-I) signaling, was independently identified as the most prominent upregulation due to the severe anthracycline cardiotoxicity by both our LC-MS setups. Furthermore, other members of the same pathway were concomitantly found to be induced (such as IFIT2, IFIT3, OAS2, OAS3, or STAT1). Interestingly, our data are in line with the recent findings obtained by other approaches suggesting the mechanistic role of this pathway in the development of the cardiotoxic phenotype.^55,56^

Indeed, our study is likely the first to describe profound and very complex remodeling of individual components of the extracellular matrix in the severe anthracycline cardiotoxicity, which is in line with the histological picture of the analyzed myocardium in our study. The results showed relatively good coverage of these anthracycline-induced changes and it was indeed a marked improvement in comparison with our previous 2D-DIGE analysis,^11^ where poor sensitivity but often also limitations related to physicochemical properties of these proteins played a considerable adverse role. However, both μLC and nLC approaches revealed only minor changes in collagenous proteins (only type VI and type XII collagens were found induced), but neither analysis revealed alterations in type I and type III collagen proteins typically involved in the pathological remodeling of the heart. Although this may appear biologically surprising, it is likely a result of the poor solubility of these proteins in a universal lysis buffer used for this analysis. Indeed, specific approaches for dedicated proteomic analysis of extracellular matrix proteins have been developed to overcome these issues,^57^ but in the present study, we aimed on global proteome rather than its specific fractions. However, our analysis revealed upregulation of numerous proteins involved in regulation of the collagen structure and function using both LC-MS/MS setups (e.g., P3H3, P4HA1, P4HA2, PLOD1, PLOD2, and LOXL2). These changes in the extracellular matrix corresponded with dramatic alterations in cellular junction proteins, particularly the upregulation of the integrins and switching of the expression of talin isoforms. On the other hand, the observed deficiency of the key components of the dystroglycan complex (e.g., downregulation of dystrophin) may imply disruption of the costamere structure and/or function. Furthermore, the analysis described marked deficiency of multiple proteins with the key role for structure of function of intercalated discs, i.e., the complex structures that connect adjacent cardiomyocytes. The latter finding may correspond with conspicuous damage to the intercalated disc reported in anthracycline-treated rabbit hearts previously by others using electron microscopy.^58^

By contrast, both LC-MS/MS setups proved the downregulation of proteins playing a role in the ribonucleotide biosynthetic process, mainly due to the decrease in ATP producing machinery with a clear drop of complex V subunits of the respiratory chain (Figure 4E). The latter finding corresponded with severe downregulation of the subunits of respiratory complex I (Figure 4D). Marked perturbation to the respiratory chain ranks among typical features of anthracycline cardiotoxicity, and it has been explained by redox cycling of anthracyclines at complex I with subsequent local damage and/or by transcriptional downregulation of mitochondrial biogenesis.^59,60^ A significant drop of the number of mitochondrial proteins observed in our study (Figure 4F) corresponds with apparent damage to mitochondria structure and function typically described in anthracycline cardiotoxicity.^11,58−60^ The substantial decrease in proteins of cardiac sarcomeres such as MYH6, MYH7, ACAC1, or TNN corresponds with the loss of myofibrils in cardiomyocytes, which is a typical histopathological hallmark of anthracycline cardiotoxicity.^58,59^ Besides changes in high abundant and often structural proteins, both LC-MS analyses allowed assessment of many low molecular protein targets including different signaling molecules (e.g., SIRT3, SIRT 5, CAMK2G, CAMK2D, PRKCD, PDK1, PDK2, PDK3, RRM2B, and PRKACB or multiple subunits of AMPK like PRKAA2, PRKAB2, and PRKAG2) with potentially interesting mechanistic role in this type of cardiac pathology.

## Conclusions

In conclusion, the performed proteomic analysis of severe anthracycline-induced cardiomyopathy proved that μLC-MS/MS is a sound and robust alternative to nLC-MS/MS. Both μLC-MS/MS and nLC-MS/MS analysis described a significant change in approximately 55% of all detected proteins with a good correlation between the data obtained by both approaches. Most of these alterations were related to proteins of cardiomyocyte sarcomeres, costameres, intercalated discs, mitochondria, and extracellular matrix. Furthermore, both analyses revealed distinct alterations in immune and defense response with a remarkable involvement of type I interferon signaling that has been recently hypothesized to be essential for anthracycline cardiotoxicity pathogenesis. Our results show that the total number of detected and significantly changed proteins can be moderately higher in nLC-MS/MS than in μLC-MS/MS analysis of myocardial proteome with severe cardiotoxicity phenotype. However, the μLC-MS/MS approach enables a less compressed and more accurate determination of TMT-based protein intensity ratios, resulting in a broader distribution of protein fold-changes compared to nLC-MS/MS.

## Data Availability

The mass spectrometry proteomic data and MaxQuant output text files have been deposited to the ProteomeXchange Consortium via the PRIDE^61^ partner repository with the dataset identifier PXD047014.

## Supporting Information Available

Supporting data for μLC-MS/MS and nLC-MS/MS (XLSX)
Additional experimental details, tables, and figures; (Table S1) urea-assisted protein digestion conditions; (Table S2) samples of the 2 species proteome model for TMT and LFQ method assessment; (Table S3) high-pH RP fractionation table, 8 fractions; (Table S4) high-pH RP fractionation table, 24 fractions; (Figure S1) impact of 2D chromatography separation on the number of the quantified proteins; (Figure S2) quantitative characteristics of single-shot DDA LC-MS/MS of 6 rabbit myocardium samples; (Figure S3) assessment of numbers of quantified proteins for first and second replicates analyzed either with μLC-MS/MS or nLC-MS/MS analysis (PDF)
