# Supplementary material for: Methodological Aspects of μLC-MS/MS for Wide-Scale Proteomic Analysis of Anthracycline-Induced Cardiomyopathy
Source: ACS Omega. 2025 Mar 18;10(12):11980–93. doi: 10.1021/acsomega.4c09377 (PMC11966270; doi:10.1021/acsomega.4c09377)
Supplement: Supplementary file 2 — ao4c09377_si_002.pdf [file ao4c09377_si_002.pdf]

Supporting Document to Original Research Article

Methodological aspects of μLC-MS/MS for wide-scale proteomic analysis of anthracycline-induced cardiomyopathy

Rudolf Kupčík[1], Olga Lenčová[2], Yvona Mazurová[2], Martin Štěrba[2] and Marie Vajrychová[1*]*

[1]Biomedical Research Centre, University Hospital Hradec Králové, Hradec Králové, Czech Republic

[2]Department of Pharmacology, Faculty of Medicine in Hradec Králové, Charles University, Hradec Králové, Czech Republic

KEYWORDS
anthracycline cardiotoxicity, 2D liquid chromatography, proteomics, mass spectrometry, nanoflow/microflow liquid chromatography

SUPPORTING METHODS

SM1: Urea-assisted in-solution protein digestion

Three 100 µg aliquots of lysate of mouse B lymphoblasts (P3X63Ag8.653, CRL-1580, ATCC, Manassas, VA, USA) were dissolved in 100 mM TEAB with 0.1% SDC and 0.8 M UREA and one aliquot in 100 mM TEAB with 0.1% SDC. To reduce disulfide bonds, samples were incubated for 60 min with 5mM TCEP at 25 °C, 37 °C or 60 °C according to Table S1. Subsequently, proteins were alkylated using 10 mM MMTS for 10 min at RT to covalently modify cysteine SH-groups. Proteins were digested using rLys-C (Wako) and sequencing grade trypsin (Promega) overnight at 1:50 enzyme-to-substrate ratio (wt/wt). Temperature for digestion was set either at 37 °C or at 25 °C according to Table S1. Digested samples were acidified by the addition of TFA to reach pH ≤2. Acid precipitated SDC was removed by phase-transfer extraction in water-saturated ethyl acetate[1] and dried by vacuum centrifugation.

Table S1 – Urea-assisted protein digestion conditions.

Sample aliquot no.	Reduction temp. (°C)	Digestion temp. (°C)	UREA concentration (M)
1	60	37	0
2	60	37	0.8
3	37	25	0.8
4	25	25	0.8

SM2: Tandem Mass Tagg labeling of peptides

The TMT-labeling experiments were performed with TMT isobaric labeling reagents (Thermo Fisher Scientific) according to manufacturer´s instructions including acetone precipitation. TMT 6-plex was used for initial TMT experiments. Two 100 µg aliquots of lysate of mouse B lymphoblasts were dissolved in 100 mM TEAB with 0.1% SDC. To reduce disulfide bonds, samples were incubated for 60 min with 5mM TCEP at 60 °C and subsequently alkylated using 10 mM MMTS for 10 min at RT to covalently modify cysteine SH-groups. Reduced and alkylated sample was precipitated in 6 vol of ice-cold acetone, kept for 4h at -20 °C and centrifuged at 8,000 × g (10 min, 4 °C). Pellet was, after decantation and drying of acetone, re-dissolved in 100mM TEAB/0.1% SDC. Proteins were digested using rLys-C (Wako) and sequencing grade trypsin (Promega) digestion at 37 °C overnight at 1:50 enzyme-to-substrate ratio (wt/wt). Digested sample was acidified by the addition of TFA. Acid precipitated SDC was removed by phase-transfer extraction in water-saturated ethyl acetate[1] and dried by vacuum centrifugation. The acetone-precipitated protein pellets (50 µg) were re-suspended with 50 µl of **50mM TEAB** and was combined with 20.5 µl (400 ug) of its respective 6-plex TMT reagent to get TMT-to-peptide ratio 8:1 (wt/wt) or with 20.5 µl of 2x diluted (200 ug) 6-plex TMT reagent to get TMT-to-peptide ratio 4:1 (wt/wt). Due to under-labeling of peptides in both ratios, TMT 6-plex isobaric labeling reagents kit was replaced by TMT 10-plex, where **100mM TEAB** is recommended for acetone-precipitated

protein pellets prior to TMT labeling and both TMT-to-peptide ratios have been proven as efficient. However, the labeling method described as above was kept for further analysis.

SM3: Preparation of mixed proteome model

S. pneumoniae[2]and mouse B lymphoblasts (P3X63Ag8.653, CRL-1580, ATCC, Manassas, VA, USA) proteins were both precipitated using ice-cold acetone. 150 μl of both sample types were individually mixed with 6 volumes of ice-cold acetone (-20 °C) and incubated for 4 h at -20 °C and centrifuged at 8,000 × g (10 min, 4 °C). After decantation of supernatant and drying of pellet, proteins were re-dissolved in 1% SDC and sonicated in a water bath for 10 min. Total protein concentration was determined by bicinchoninic acid and copper sulfate solution (BCA protein assay kit). Sample volume corresponding to desired protein amount specified in Table S2 were mixed together. Every individual protein mixture (A-E) was subsequently mixed with 1M TEAB and 1% SDC to get final concentration of 100 mM TEAB and 0.1% SDC, respectively. Cysteine bonds were reduced by incubation of protein mixture and incubated with 5 mM TCEP for 60 min at 60 °C. Proteins were then alkylated with 10 mM MMTS for 10 min at RT to covalently modify cysteine SH-groups. Proteins were digested using rLys-C (Wako) and sequencing grade trypsin (Promega) at 37 °C overnight at 1:50 enzyme-to-substrate ratio (wt/wt). Digested samples were acidified by the addition of trifluoroacetic acid (TFA) to reach pH ≤2. Acid precipitated SDC was removed by phase-transfer extraction in water-saturated ethyl acetate[1]and dried by vacuum centrifugation.

Table S2 – Samples of 2 species proteome model for TMT and LFQ methods assessment.

Sample	*S. Pneumoniae* (μg)	Mouse lymphoblast B cells (μg)
A	50	50
B	25	50
C	12.5	50
D	10	50
E	37.5	50
F	5	50

Peptide mixtures were reconstituted in 100 mM TEAB (pH 8.5) and split into 2 parts – 1st part (for LFQ) was desalted using Discovery DSC-18 SPE (Sigma-Aldrich) RP solid-phase extraction cartridges, aliquoted, dried by vacuum centrifugation and stored at -80 °C for nanoLC-MS/MS analysis. 2nd part of peptide mixture samples was combined with its respective 10-plex TMT reagent to get TMT-to-peptide ratio 4:1 (wt/wt).[3] Samples A-E were sequentially derivatized with 127C, 128N, 128C, 129N, 129C and 130N m/z TMT labels. The peptide–TMT mixture was incubated for 1 h at 25 °C and 500 rpm, and the labeling reaction was quenched by addition 8 μl of 5% hydroxylamine and incubated for 15 min at 25 °C and 500 rpm. Subsequently, TMT labeled samples were combined and desalted using Discovery DSC-18 SPE (Sigma-Aldrich) RP solid-phase extraction cartridges, aliquoted, dried by vacuum centrifugation and stored at -80 °C for nanoLC-MS/MS analysis.

SM4: nLC-MS/MS – label free approach

Non-fractionated samples were analyzed in two technical replicates. The separation of peptides for nLC-MS/MS analysis was done using the UltiMate 3000 RSLCnano system (Thermo Scientific, Bremen, Germany). The analytical system consisted of PepMap100 C18, 3 µm, 100 Å, 75 µm × 20 mm trap column and PepMap RSLC C18, 2 µm, 100 Å, 75 µm × 250 mm analytical column (both from Thermo Scientific). The samples were dissolved in loading solvent (2% AcN/0.1% TFA) and loaded onto the trap column for 5 min at a flow rate 5 µL/min. The separation was performed by 240 min linear segmented gradient running from 2% B to 9% B in 57 min followed by 9% B to 34.5% B in 160 min and by 34.5% B to 45% B in 23 min (2% ACN/0.1% FA as phase A; 80% AcN/0.1% FA as phase B) at a flow rate of 250 nL/min. Eluted peptides were analyzed with Q-Exactive Plus connected to LC system via Nanospray Flex ion source (both Thermo Scientific, Bremen, Germany). Positive ion full scan MS spectra were acquired on Orbitrap with setup specified in Table 1.

SM5: µLC-MS/MS – label-free approach

Non-fractionated samples were analyzed in two technical replicates. The separation of peptides for µLC-MS/MS analysis was done using the UltiMate 3000 binary RSLC system (Thermo Scientific, Bremen, Germany). The system was configured for µLC analysis according to previously published article.[4] The analytical system was then equipped with HALO Peptide ES-C18, 2.7 µm, 160 Å, 1.0 mm × 250 mm analytical column (Advanced Materials Technology). The samples were reconstituted in mobile phase A and the separation was performed by linear gradient running from 0.2% to 45% of mobile phase B (3% DMSO/0.4% acetic acid (HAc)/0.1% FA as phase A; 78% AcN/3% DMSO/0.4% HAc/0.1%FA as phase B) for 250 min at a flow rate of 68 µL/min. Eluted peptides were introduced into Q-Exactive Plus mass spectrometer via EASY-Spray ion source equipped with HESI-II probe (all Thermo Scientific, Bremen, Germany). The settings of MS analysis were optimized in order to find best performance with respect to highest number of quantified proteins. In our case the best performance was found for positive ion full scan MS spectra acquired on Orbitrap with setup specified in Table 1.

SM6: Data evaluation

All acquired MS and MS/MS data were processed in by MaxQuant v1.6.14.0 and with its built-in search engine, Andromeda. For label-free data processing, standard type with multiplicity 1 and LFQ with min. ratio count 2 was selected in group specific parameters. All proteins were filtered out for at least two valid values across three technical replicates.

SM7: Basic chromatography fractionation for 2D TMT LC-MS/MS analysis

Dried TMT-labeled peptides (120 µg) were re-dissolved in mobile phase A (2% AcN/10 mM NH_4FA) and injected in XBridge BEH C18 column (2.5 µm, 2.1 µm x 150 mm; Waters) on UltiMate 3000 RSLC system (Thermo Scientific, Bremen, Germany). In the first strategy, individual fractions (45 s) were collected in segmented gradient of mobile phase B (80% AcN/10 mM NH_4FA) at flow rate of 0.3 mL/min. Gradient was running to 2% B in 2 min, from 2% B to 20% B in 8 min, followed by 20% B to 50% B in

36 min and by 50% B to 52% B in 5 min. All fractions were collected into a polypropylene 96 well plate (Agilent Technologies, Santa Clara, CA, USA) starting from 4 min to 52 yielding 64 fractions. Collected fractions were pooled into 8 fractions (Table S3) dried by vacuum centrifugation and stored at -80°C. Collected fractions were further re-dissolved in loading solvent (2% AcN/0.1% TFA), loaded in trap column at flow rate of 5 µL/min for 5 min. Peptides were separated using linear gradient (2% ACN/0.1% formic acid (FA) as phase A; 80% ACN/0.1% FA as phase B), running from 2 to 9% B in 57 min, from 9 to 34.5% B in 160 min, and from 34.5 to 45% B in 23 min at flow rate of 250 nL/min. Eluted peptides were introduced into Q-Exactive Plus mass spectrometer via Nanospray Flex ion source (both Thermo Scientific, Bremen, Germany). Positive ion full scan MS spectra were acquired using parameters specified in the main manuscript (Table 1). All fractions/samples were analyzed in two technical replicates. The second strategy (24 fraction-method, Table S4) followed by µLC-MS/MS and nLC-MS/MS is described in the main manuscript in detail.

Table S3: High-pH RP fractionation table – 8 fractions.

**High-pH RP fraction no.**								**Combined fractions** (from 8 fractions on the left)
8	16	24	32	40	48	56	64	H
7	15	23	31	39	47	55	63	G
6	14	22	30	38	46	54	62	F
5	13	21	29	37	45	53	61	E
4	12	20	28	36	44	52	60	D
3	11	19	27	35	43	51	59	C
2	10	18	26	34	42	50	58	B
1	9	17	25	33	41	49	57	A

Table S4: High-pH RP fractionation table – 24 fractions.

**High-pH RP fraction no.**	1	2	3	4	5	6	7	8	9	10	11	12
	25	26	27	28	29	30	31	32	33	34	35	36
	49	50	51	52	53	54	55	56	57	58	59	60
**Combined fractions** (from 3 fractions above)	A	B	C	D	E	F	G	H	CH	I	J	K
**High-pH RP fraction no.**	24	23	22	21	20	19	18	17	16	15	14	13
	48	47	46	45	44	43	42	41	40	39	38	37
	72	71	70	69	68	67	66	65	64	63	62	61
**Combined fractions** (from 3 fractions above)	W	V	U	T	S	R	Q	P	O	N	M	L

SUPPORTING FIGURES

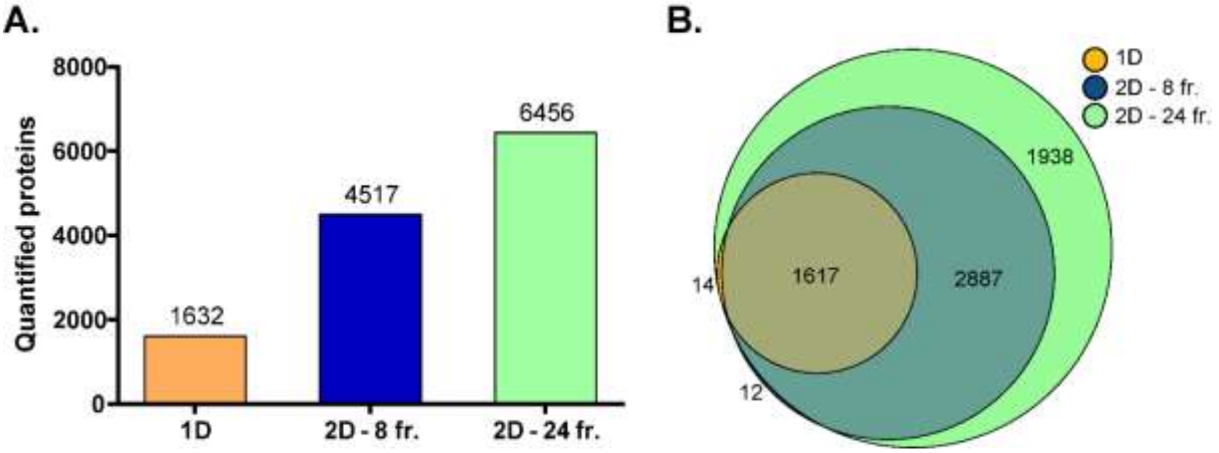

Figure S1: *The impact of 2D chromatography separation on the number of the quantified (A) and the newly identified (B) proteins.*

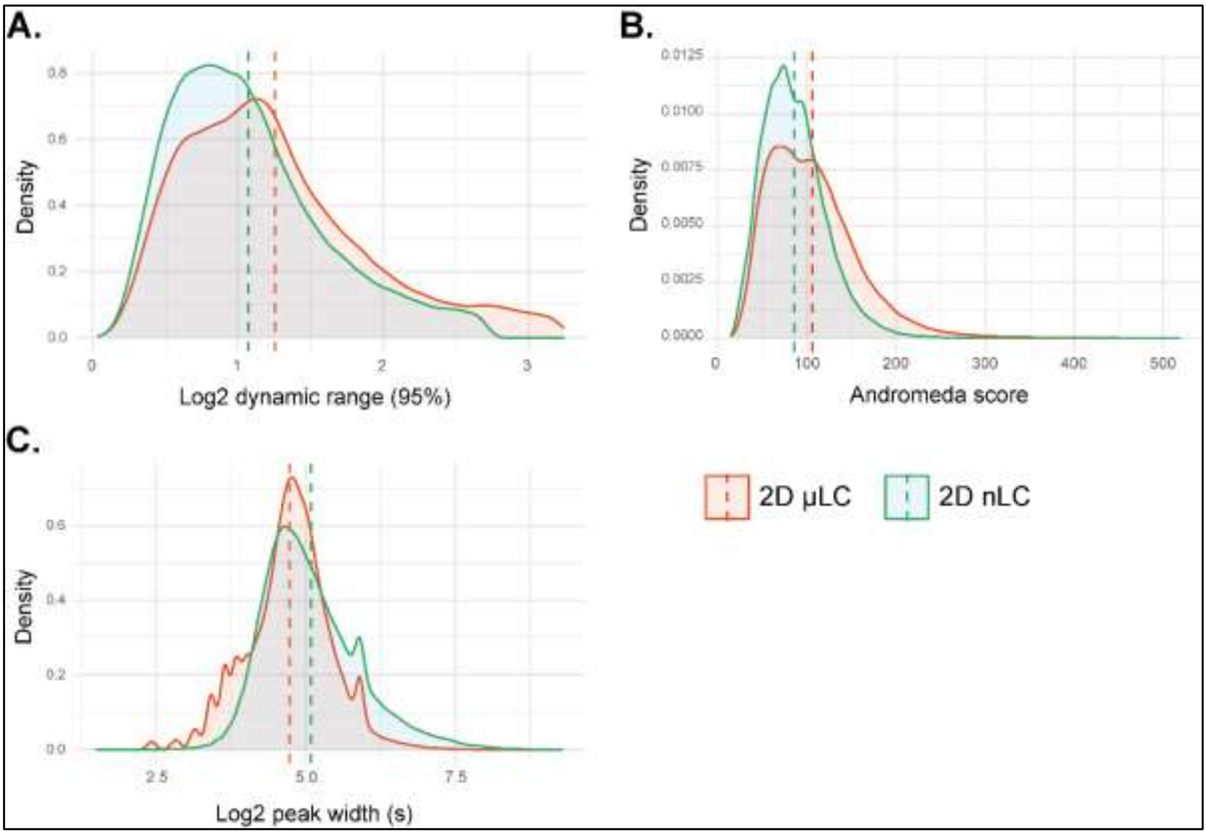

Figure S2: *A quantitative characteristics of single-shot DDA LC-MS/MS of 6 rabbit myocardium samples. A) Dynamic range, B) PSM probability based on Andromeda score, C) (log_2) overall peak width.*

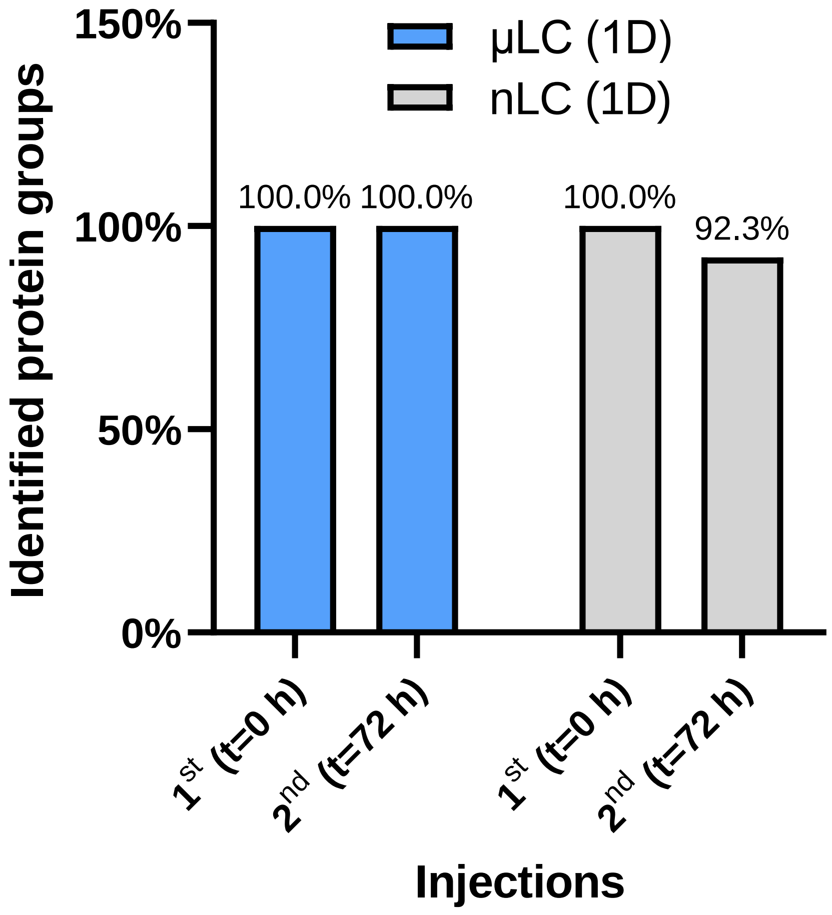

Figure S3: *Assessment of numbers of quantified proteins for first and second replicates analyzed either with μLC-MS/MS or nLC-MS/MS analysis.*

Number of quantified proteins using 1D nLC dropped by 7.7% between replicates, whereas μLC analysis kept number of quantified proteins at the same level. Time period between replicates was ≈ 72h.

REFERENCES:

(1) Lin, Y.; Liu, Y.; Li, J.; Zhao, Y.; He, Q.; Han, W.; Chen, P.; Wang, X.; Liang, S. Evaluation and optimization of removal of an acid-insoluble surfactant for shotgun analysis of membrane proteome. *Electrophoresis* **2010**, *31* (16), 2705-2713.

(2) Ulrych, A.; Holeckova, N.; Goldova, J.; Doubravova, L.; Benada, O.; Kofronova, O.; Halada, P.; Branny, P. Characterization of pneumococcal Ser/Thr protein phosphatase phpP mutant and identification of a novel PhpP substrate, putative RNA binding protein Jag. *BMC Microbiol.* **2016**, *16* (1), 247.

(3) Zecha, J.; Satpathy, S.; Kanashova, T.; Avanessian, S. C.; Kane, M. H.; Clauser, K. R.; Mertins, P.; Carr, S. A.; Kuster, B. TMT Labeling for the Masses: A Robust and Cost-efficient, In-solution Labeling Approach. *Mol. Cell. Proteomics* **2019**, *18* (7), 1468-1478.

(4) Lenco, J.; Vajrychova, M.; Pimkova, K.; Proksova, M.; Benkova, M.; Klimentova, J.; Tambor, V.; Soukup, O. Conventional-Flow Liquid Chromatography-Mass Spectrometry for Exploratory Bottom-Up Proteomic Analyses. *Anal. Chem.* **2018**, *90* (8), 5381-5389.
